# P-1092. What Do Patients Think About Contact Precautions? A Survey of 70 Patients with Methicillin-resistant Staphylococcus aureus and Multidrug-resistant Gram-negative Infections

**DOI:** 10.1093/ofid/ofaf695.1287

**Published:** 2026-01-11

**Authors:** Shatha AlShanqeeti, Matthew B Goetz, Kevin Ikuta, Gio Baracco Lira, Christopher J Crnich, Mary Bahr-Robertson, Lyndsay M O’Hara, Anthony Harris

**Affiliations:** University of Maryland, Baltimore, MD; VA Greater Los Angeles Healthcare System, Los Angeles, California; West Los Angeles VA, Los Angeles, California; Miami VA Healthcare System, Miami, Florida; University of Wisconsin School of Medicine and Public Health, Madison, WI; University of Maryland Baltimore, Baltimore, Maryland; University of Maryland School of Medicine, Baltimore, Maryland; University of Maryland School of Medicine, Baltimore, Maryland

## Abstract

**Background:**

The topic of contact precautions remains controversial. Risk-tailored contact precautions, e.g. requiring gowns and gloves only for “high risk” healthcare workers or those touching the patient, have been proposed as an alternative. Despite its wide use, few studies have explored patients’ perspectives towards contact precautions. The aim of this study was to describe hospitalized patients’ opinions and preferences related to the use of contact precautions for MRSA and multidrug-resistant gram-negative infections.

**Table 1:**
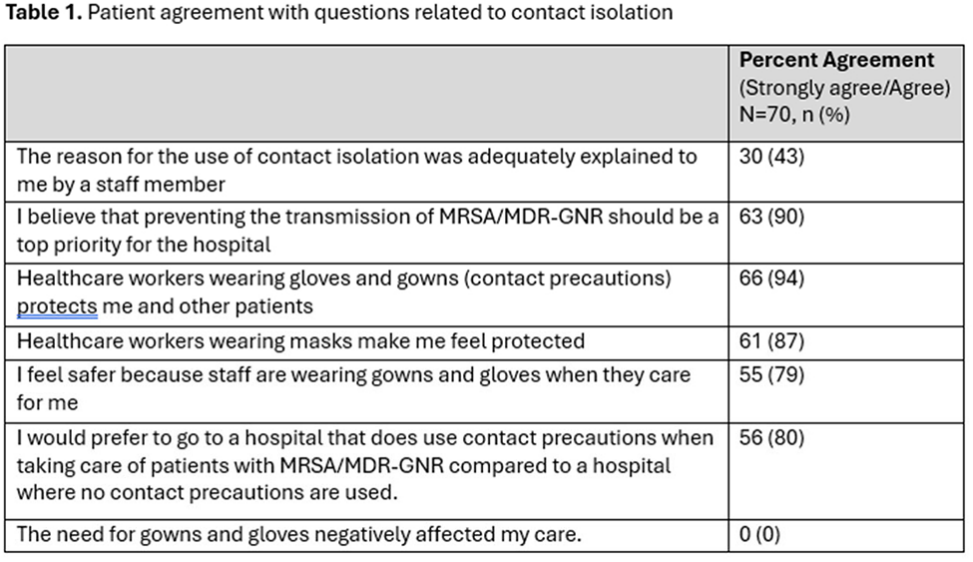
Patient agreement with questions related to contact isolation

**Figure 1:**
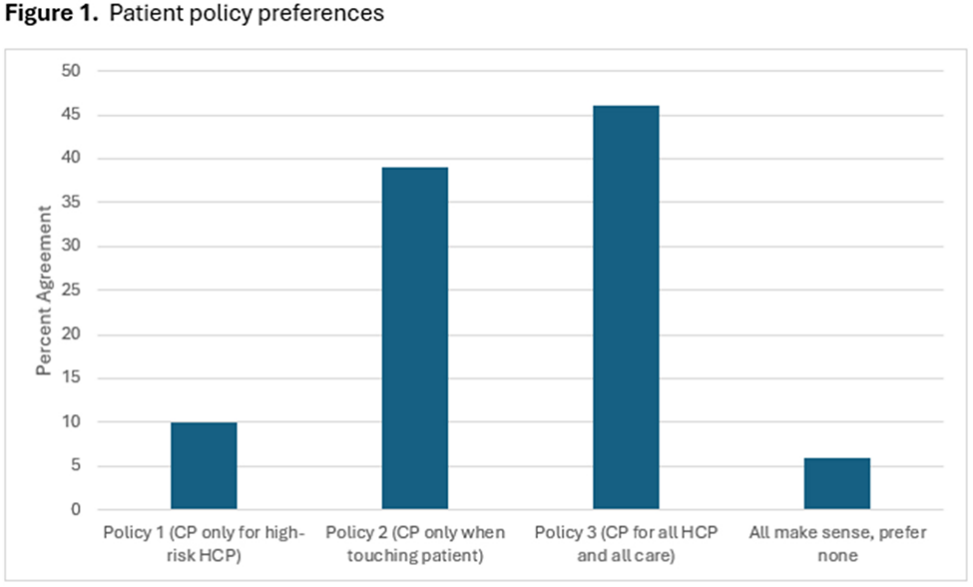
Patient policy preferences

**Methods:**

A cross-sectional survey was administered to 30 adult patients with MRSA and 40 with multidrug-resistant gram-negative colonization or infection at two hospitals in Baltimore, MD. The survey was comprised of eleven questions, in which most utilized a 7-point Likert scale and assessed the patient’s opinions on the following topics: (1) effectiveness of contact precautions, (2) patient preference of hospital based on presence or absence of contact precautions policy, (3) adequacy of explanation of reason for contact precautions by hospital staff, (4) preference of mask wearing by healthcare workers, and (5) agreement with two proposed alternative risk-based policies.

**Results:**

As shown in Table 1, most patients surveyed (60, 90%) believe that preventing the transmission of MRSA and MDR-GN should be a top priority for hospitals and 66 (94%) feel that healthcare workers wearing gloves and gowns protect them and other patients. None reported that the need for gloves and gowns negatively affected their care. When asked to choose between the two risk-based policies and a policy where healthcare workers wear gloves and a gown all the time, 7 (10%) preferred Policy 1 (certain healthcare workers), 27 (39%) preferred Policy 2 (touching the patient), 32 (46%) Policy 3 and 4 (6%) reported that all policies make sense.

**Conclusion:**

This study suggests that some patients support a risk-based approach to isolation policies, but these policies are not necessarily preferred over the policy of all healthcare workers wearing contact precautions all the time. Patients believe that contact precautions protect them and other patients, and do not believe that contact precautions have a negative impact on their care.

**Disclosures:**

Christopher J. Crnich, MD, PhD, Merck: Grant/Research Support Anthony Harris, MD, MPH, UpToDate: Advisor/Consultant

